# Effect of Probiotic *Streptococcus salivarius* K12 Application on Palatal Wound Healing: An in Vivo Study

**DOI:** 10.21315/mjms-09-2024-657

**Published:** 2025-04-30

**Authors:** Nissia Ananda, Dwi Ariawan, Vera Julia, Vetnizah Juniantito, Kania Alishaputri Wibisono, Endang Winiati Bachtiar

**Affiliations:** 1 Dental Department, Rumah Sakit Universitas Indonesia, Depok, Indonesia; 2 Faculty of Dentistry, Universitas Indonesia, Jakarta, Indonesia; 3 Department of Oral and Maxillofacial Surgery, Faculty of Dentistry, Universitas Indonesia, Jakarta, Indonesia; 4 Department of Veterinary Clinic, Reproduction and Pathology, Faculty of Veterinary Medicine, IPB University, Bogor, Indonesia; 5 Department of Oral Biology, Faculty of Dentistry, Universitas Indonesia, Jakarta, Indonesia

**Keywords:** inflammation, probiotics, Streptococcus salivarius, wound healing

## Abstract

**Background:**

Oral and maxillofacial surgeons often deal with skin wounds, where the healing process involves phases such as inflammation and remodelling. Excessive inflammation can disrupt healing, leading to complications such as abnormal scarring and impaired tissue regeneration. Probiotics, especially strains such as *Streptococcus salivarius* K12, show promise in modulating inflammation and enhancing wound healing. Recent studies have aimed to explore how these probiotics affect inflammatory markers during wound healing, potentially offering new therapeutic benefits.

**Methods:**

This quasi-experimental study, conducted in June–July 2023 at IPB (Institut Pertanian Bogor) University’s Veterinary Teaching Hospital, included 24 healthy Sprague-Dawley rats. The rats were randomly assigned to two groups: one was treated with *Streptococcus salivarius* K12, and the other was the control group. The treated group’s palatal wounds received *Streptococcus salivarius* K12, covered with a daily-changed mucosal patch, whereas the control group received only the patch. Four rats per group were sacrificed on days 3, 7, and 14. Wound areas were examined histopathologically, and neutrophil, macrophage, and lymphocyte counts were quantified using ImageJ software. Statistical analysis was conducted using MANOVA and the Tukey HSD test.

**Results:**

All inflammatory indicators (neutrophils, macrophages, and lymphocytes) showed significant differences between the control and treated groups, as well as across different necropsy days.

**Conclusions:**

The inflammation modulation by the probiotic *Streptococcus salivarius* K12 contributes to enhanced wound healing. In the treated group, there were higher counts of neutrophils, macrophages, and lymphocytes compared with the control group, with notable variations observed over time.

## Introduction

Oral and maxillofacial surgeons frequently encounter patients with skin wounds, initiating a complex wound healing process. This intricate process aims to restore the anatomical and physiological integrity of the injured tissues and progresses through phases such as hemostasis, inflammation, proliferation, and remodelling. (1) Research has shown that excessive inflammation can lead to several disadvantages in the wound healing process. First, it can disrupt the normal course of wound healing, leading to complications such as abnormal scar formation; this dysregulated inflammatory response may impede wound closure, hinder tissue regeneration, and contribute to the development of hypertrophic scars, keloids, and fibrotic scar tissue (2, 3). Second, excessive inflammation can exacerbate tissue damage, hinder cell migration and proliferation, and disturb the deposition of crucial extracellular matrix components required for wound healing (4, 5); this chronic inflammatory state in non-healing wounds perpetuates a destructive cycle of tissue damage and inflammation, further impeding the progression of the healing process (5). Therefore, maintaining a delicate balance of the inflammatory response, including the involvement of neutrophils, macrophages, and lymphocytes, throughout all periods of the proliferation phase is essential for effective wound healing.

Probiotics, which are living and viable microorganisms, confer benefits to the host when consumed in adequate amounts. Acknowledged under Generally Recognised as Safe (GRAS) status, these exogenous microorganisms demonstrate a wide array of advantageous properties that impact various aspects of human health. In the field of clinical dentistry, probiotic therapy has gained traction as an adjunct treatment option in recent years; this approach harnesses the potential of probiotics to promote oral health and address specific dental concerns, offering a complementary avenue for managing oral conditions and supporting overall well-being (6). Probiotics have also emerged as promising agents in the field of wound healing, demonstrating their ability to modulate the inflammatory response and foster tissue repair. Researchers have explored topical probiotic applications, aiming to regulate inflammation, prevent infections, and optimise wound healing outcomes (7, 8). For instance, a study revealed that a combination of *Streptococcus salivarius* K12 and *Streptococcus salivarius* M-18 could suppress the production of the pro-inflammatory cytokines IL-6 and IL-8, highlighting the anti-inflammatory potential of these strains (9).

Although research on *Streptococcus salivarius* in wound healing is still limited, lactic acid bacteria (LAB) serve as a compelling example of how probiotics can positively impact the healing process. Studies on LAB suggest their potential role in enhancing wound healing, possibly through mechanisms such as immune modulation and the upregulation of neuropeptide hormones such as oxytocin. This growing body of evidence on LAB highlights the broader potential of probiotics in wound management, setting a promising foundation for exploring *Streptococcus salivarius* and other species as therapeutic agents in wound care (10).

Therefore, the specific impact of *Streptococcus salivarius* K12 administration on inflammation parameters such as neutrophils, macrophages, and lymphocytes remains unclear. Hence, this study aimed to elucidate the effects of employing the probiotic *Streptococcus salivarius* K12 on these inflammation markers during the healing process of palate wounds. Through this investigation, we aimed to gain deeper insights into the potential therapeutic benefits of probiotic interventions in the context of wound healing.

## Methods

This study employed a quasi-experimental design; the subjects were recruited from June 2023 to July 2023 at the Veterinary Teaching Hospital, School of Veterinary Medicine and Biomedicine, IPB University, Bogor.

### Rats

Sprague-Dawley rats were housed in the Experimental Animal Cage Unit of the LPPMI-PB Biopharmaceutical Study Centre. The study included 24 healthy white Sprague-Dawley rats weighing between 200 and 300 g and aged 8 weeks. Rats that became ill or died during the research were excluded. The rats were randomly assigned to two distinct groups: the treated group, which received treatment with BLIS K12^®^, and the control group.

### Wound Model

Anaesthesia and a muscle relaxant were administered using Ketamine–Hameln at a dose of 95 mg/kg body weight (BW) and Xylazine at a dose of 5 mg/kg BW. Then, the palates of the rats were sterilised using sterile gauze, tweezers, and 70% alcohol. After sterilisation, a punch biopsy was performed at the midline, creating a circular wound with a diameter of 5 mm. The probiotic *Streptococcus salivarius* K12 was ground using a mortar and pestle and then applied to the rat’s wound, followed by the placement of a mucosal patch over it. In the control group, only a mucosal patch was placed on the wound. The mucosal patch used contained no active ingredients, ensuring that it did not introduce bias when testing the active substance (probiotic) in this study. Treatment was administered daily throughout the experimental period, and the mucosal patch was changed daily. Four subjects from each group were sacrificed on days 3, 7, and 14.

### Wound Healing Observation

The wound areas of the necropsed subjects were fixed using Bouin’s solution. Histopathological examination was conducted using hematoxylin and eosin staining. Quantitative measurements of the numbers of neutrophils, macrophages, and lymphocytes were performed using ImageJ software.

### Statistical Analysis

The statistical analysis of the data was conducted using IBM SPSS Statistics 25. The normality of data distribution was assessed using the Shapiro–Wilk test. Comparative tests were performed using MANOVA; the Tukey honestly significant difference (HSD) test was used for the post hoc analysis.

## Results

The mean neutrophil, macrophage, and lymphocyte values are presented in Table 1. This research results indicate that neutrophils, macrophages, and lymphocytes were generally more numerous in the treated group (wounds treated with probiotic *Streptococcus salivarius* K12) compared with the control group. Additionally, neutrophil counts decreased from day 3 to day 7 and further to day 14 in both groups. In contrast, macrophages and lymphocytes peaked on day 7, aligning with the middle of the proliferation phase. The highest neutrophil count was observed in the treated group on day 3, whereas the lowest was observed in the control group on day 14. For macrophages, the peak count occurred in the treated group on day 7 and was lowest in the control group on day 3. Similarly, lymphocyte counts were highest in the treated group on day 7 and lowest in the control group on day 3.

The residuals were tested for normality using the Shapiro–Wilk test, which confirmed a normal distribution (*P* > 0.05). Then, Box’s M test was conducted to evaluate the homogeneity of the covariance matrices; the results showed no significant differences (*P* > 0.05), thereby supporting the homogeneity assumption. Multicollinearity among the dependent variables (neutrophils, macrophages, and lymphocytes) was also assessed; the correlation coefficients were found to be within acceptable limits, indicating no issues with multicollinearity. Finally, Levene’s test for equality of variances was performed, with the results showing no significant differences (*P* > 0.05), thus meeting the assumption of homogeneity of variances. Therefore, we used MANOVA to analyse the data. The MANOVA results (Table 2) revealed that all inflammatory indicators (neutrophils, macrophages, and lymphocytes) differed significantly between the control and treated groups, as well as across different necropsy days. Furthermore, the interaction between the groups and necropsy days was significant only for the lymphocyte group.

Because there were significant differences in neutrophils, macrophages, and lymphocytes across necropsy days, as indicated by the MANOVA test, we performed a post hoc test using the Tukey HSD. The results of the Tukey HSD post hoc test are presented in Table 3. The results indicate that neutrophils and lymphocytes had significantly different counts between all days (days 3 and 7, days 7 and 14, and days 3 and 14). For macrophages, significant differences were observed between days 3 and 7 and between days 7 and 14. However, the number of macrophages did not differ significantly between days 3 and 14.

## Discussion

The proliferation phase of wound healing is marked by re-epithelialization, neovascularization, and extracellular matrix deposition (11). Transitioning from the inflammatory phase to the proliferation phase is crucial for efficient tissue repairing and remodelling (12). Neutrophils play a crucial role in the early stages by clearing debris and preventing infection; however, their excessive activity can prolong inflammation and delay healing (13, 14). Proper clearance of neutrophils by macrophages is essential for resolving inflammation and advancing to the proliferative phase (15, 16). Regulation of neutrophil recruitment is important for this transition (17). Neutrophils initiate debridement and combat microbes by releasing Neutrophil Extracellular Traps (NET), which are web-like structures composed of DNA and antimicrobial proteins. However, excessive NET formation can delay healing, particularly in conditions such as diabetic foot ulcers, and contribute to inflammation, autoimmunity, and thrombosis (18, 19). Effective neutrophil function is crucial for timely wound healing (20). During the proliferative phase, neutrophils stimulate the release of growth factors and cytokines, aiding tissue repair and regeneration (21); their uptake by macrophages signals inflammation resolution, allowing progression to the healing phase (22).

In this study, we observed that neutrophil counts decreased from days 3 to 7 and further to day 14 in the treated and control groups. This finding aligns with the understanding that neutrophil counts decline over time to prevent excessive inflammation and delay wound healing (13, 14). Day 3 was used to represent the early proliferation phase, which overlaps with the inflammation phase; during this period, we found the highest number of neutrophils (1). This supports the notion that neutrophils are most abundant in the early stages of wound healing to clear debris and prevent infection; however, their numbers decrease as the healing process progresses to ensure proper tissue repair and reduce the risk of scarring. In contrast, the application of the topical probiotic *Streptococcus salivarius* K12 to palatal wounds increased neutrophil counts. Despite this increase, no significant interaction effect was observed between the probiotic application and the necropsy days. This suggests that although the neutrophil counts were higher in the treated group, they were still effectively regulated and decreased over time, thereby preventing excessive scar formation.

Following the neutrophil activity, macrophages play a crucial role in the proliferation phase by polarizing toward the M2 phenotype, which promotes an anti-inflammatory, pro-repair environment (23). This shift is particularly important for accelerating wound healing (24). While macrophages are important for repair, their over-activation can lead to prolonged healing phases and fibrotic tissue formation (25). Their plasticity allows them to modulate the inflammatory response and promote the transition from inflammation to proliferation (26). During this phase, macrophages release growth factors and cytokines such as VEGF, PDGF, IL-1β, IL-6, and TNF-α, which activate fibroblasts and endothelial cells, aiding tissue regeneration (27). The balance between pro-inflammatory (M1) and anti-inflammatory (M2) macrophages shapes the local wound microenvironment, with M1 and M2 dominating the inflammatory and proliferation phases, respectively (28). Macrophages also downregulate inflammation, promote angiogenesis, form granulation tissue, and deposit collagen (29). Modulating macrophage function using bioactive wound dressings and scaffolds that mimic the extracellular matrix (ECM) can enhance tissue repair by supporting the shift from chronic inflammation to the proliferative phase (30). Polarizing macrophages toward the M2 phenotype is key to facilitating tissue regeneration during the early proliferation phase (31).

The results indicate that macrophage counts peaked in the treated group on day 7, the midpoint of the proliferation phase, whereas the lowest count was observed in the control group on day 3. Macrophages play a crucial role during the proliferation phase, and their numbers typically peak at this stage. As the phase progresses, a decrease in the macrophage count is required to prevent excessive scar formation (25). Interestingly, the application of *Streptococcus salivarius* K12 increased the macrophage numbers during the early and middle proliferation phases, promoting tissue proliferation. However, by the late proliferation phase, the treated group had lower macrophage counts than the control group, suggesting that the role of the macrophages was completed earlier, potentially facilitating faster wound closure (27).

Lymphocytes, particularly B and T cells, also play a crucial role in the proliferation phase of wound healing by contributing to the immune response, tissue repair, and regeneration; however, excessive B-cell proliferation can cause aberrant immune responses and tissue damage. CD4+ T cells, regulated by umbilical mesenchymal stem cell-derived exosomes, promote balanced immune responses and tissue regeneration by inhibiting excessive proliferation and apoptosis (32). CD4+ T cells also reduce scarring and increase neovascularisation during dermal wound healing (33). The balance between lymphocyte populations is crucial for optimal tissue regeneration and scar formation (34). γδ T cells and TCR signalling are particularly crucial for epidermal wound healing functions (35).

The findings of this study indicate that the peak count of lymphocytes occurs in the treated group on day 7, corresponding to the middle period of the proliferation phase, which mirrors the results observed for macrophages. Maintaining a harmonious equilibrium among lymphocyte populations is important for achieving optimal tissue regeneration and facilitating scar formation (34). This result aligns with the theory that the transition from the proliferative phase to the maturation phase of wound healing involves the clearance of immune cells, including lymphocytes, from the wound site. The clearance of lymphocytes marks the conclusion of the acute wound healing process and sets the stage for tissue maturation and scar formation (36). The application of the topical probiotic *Streptococcus salivarius* K12 on palatal wounds leads to an increased number of lymphocytes throughout all phases of the proliferation phase—early, middle, and late— compared with the control group. Furthermore, the MANOVA test results reveal a significant effect of interaction between the treatment groups and necropsy days for lymphocyte counts. This significant finding indicates that the impact of probiotic treatment on lymphocyte counts varies across different time points during the wound healing process. This interaction highlights the importance of timing when administering probiotic treatment, emphasising its varying effects on lymphocyte levels at different proliferation stages.

This study is the first to analyse the effects of the probiotic *Streptococcus salivarius* K12 on neutrophils, macrophages, and lymphocytes during the proliferation phase of wound healing. As a pilot study using Sprague-Dawley rats, this study has limitations owing to the physiological differences between rats and humans. Therefore, further research is required to validate the effects of *Streptococcus salivarius* K12 on intraoral wounds, with larger sample sizes and human subjects to ensure that the findings are applicable to clinical settings.

## Conclusions

This study highlighted the potential of the probiotic *Streptococcus salivarius* K12 in enhancing wound healing by modulating inflammation. The treated group showed higher neutrophil, macrophage, and lymphocyte counts than the control group, with significant variations over time. Neutrophils decreased over time, preventing excessive inflammation, whereas macrophages and lymphocytes peaked around day 7, indicating their crucial roles in the proliferation phase.

## Figures and Tables

**Table 1:** Neutrophils, macrophages, and lymphocytes of both groups across various necropsy days

		Neutrophils (*n*)	Macrophages (*n*)	Lymphocytes (*n*)
Control Group	Day 3	47.20 ± 10.82	35.70 ± 10.61	20.10 ± 0.12
	Day 7	34.05 ± 3.83	49.65 ± 6.11	28.20 ± 1.57
	Day 14	15.95 ± 10.68	40.25 ± 6.12	25.00 ± 1.67

Treated Group	Day 3	68.90 ± 4.58	59.60 ± 5.19	30.50 ± 0.62
	Day 7	42.05 ± 1.53	65.05 ± 3.24	39.40 ± 1.59
	Day 14	20.65 ± 5.70	39.25 ± 6.84	29.75 ± 1.80

**Table 2:** Results of the MANOVA with regard to differences in neutrophils, macrophages, and lymphocytes between the groups and the associated necropsy days

Comparison		*F*	*P*-value
Groups	Neutrophils	15.703	0.001*
	Macrophages	18.666	< 0.001*
	Lymphocytes	244.404	< 0.001*

Necropsy days	Neutrophils	62.901	< 0.001*
	Macrophages	13.985	< 0.001*
	Lymphocytes	83.168	< 0.001*

Interaction of groups and necropsy days	Neutrophils	3.235	0.063
	Macrophages	5.858	0.011
	Lymphocytes	13.132	< 0.001*

Note:

* statistically significant

**Table 3:** Tukey’s post hoc HSD test results for inter-necropsy-day comparisons

Variable	Comparison		Sig.
Neutrophils	Necropsy day 3	Necropsy day 7	< 0.001*
		Necropsy day 14	< 0.001*
	Necropsy day 7	Necropsy day 14	< 0.001*

Macrophages	Necropsy day 3	Necropsy day 7	0.011*
		Necropsy day 14	0.154
	Necropsy day 7	Necropsy day 14	< 0.001*

Lymphocytes	Necropsy day 3	Necropsy day 7	< 0.001*
		Necropsy day 14	0.020*
	Necropsy day 7	Necropsy day 14	< 0.001*

Notes: Sig. = Significance level = *P*-value;

* statistically significant
